# The mediating role of family function in the relationship between stressful life events and depressive symptoms in mid-pregnancy women: a birth cohort study in Wuhan, China

**DOI:** 10.3389/fpsyt.2025.1588065

**Published:** 2025-09-23

**Authors:** Yihan He, Hui Zhou, Jiyu Luo, Ao Xu, Dongmei Qiu, Haiyun Huang, Xiaorui Peng, Yujia Zhou, Lingyun Xu, Yang Li, Yuanyuan Li, Xiang Hu, Hongling Zhang

**Affiliations:** ^1^ School of Medicine and Health, Wuhan Polytechnic University, Wuhan, China; ^2^ Office of Party Committee, Huangshi Hospital of Traditional Chinese Medicine, Huangshi, China; ^3^ School of Life Science and Technology, Wuhan Polytechnic University, Wuhan, China; ^4^ Key Laboratory of Environment and Health, Ministry of Education & Ministry of Environmental Protection, and State Key Laboratory of Environmental Health (Incubation), School of Public Health, Tongji Medical College, Huazhong University of Science and Technology, Wuhan, China

**Keywords:** family functioning, stressful life events, second trimester of pregnancy, depression, mediation

## Abstract

**Background:**

In current research on prenatal depression among pregnant women, the role of family functioning is crucial yet often overlooked. Specifically, in the field of maternal mental health, relatively limited attention has been given to the psychological well-being of women during the second trimester of pregnancy. This study aims to assess the depression, family functioning, and stressful life events of women in their second trimester, and to explore whether family functioning mediates the relationship between stressful events and depressive symptoms.

**Methods:**

A questionnaire survey was conducted with 3,386 pregnant women from the Wuhan Healthy Baby Birth Cohort. Participants completed the 10-item Center for Epidemiologic Studies Depression Scale (CESD-10), the Family APGAR Index, and the 18-item Stressful Life Events Scale. Structural equation modeling was applied to analyze the data.

**Results:**

The findings revealed significant interrelationships among depression, family functioning, and stressful life events. Family functioning was found to partially mediate the relationship between stressful life events and depressive symptoms during the second trimester, explaining 21.9% of the variance.

**Conclusions:**

These findings suggest that improving family functioning and fostering communication can help reduce depressive symptoms during the second trimester, supporting maternal mental health.

## 1 Introduction

Prenatal depression represents a major public health challenge worldwide (1). It increases the risk of pregnancy-related complications, affects the physical health of both pregnant women and their neonates (2), and has long-term implications for the overall development of infants and young children (3). This is particularly evident in low- and middle-income countries, where one in six pregnant women is affected by prenatal depression. In mainland China, a meta-analysis reported a prevalence of 19.7% for antenatal depression and 14.8% for postpartum depression (4). Notably, depressive symptoms during the second trimester often serve as early indicators of late-term and postpartum depression (5). Early identification and intervention for second trimester depression are therefore crucial in mitigating the progression of depressive symptoms.

Family functioning, a multidimensional construct, refers to a family’s ability to coordinate and fulfill the survival, security, and social needs of its members. It serves as a comprehensive indicator for evaluating family dynamics and task performance (6). As a key determinant of emotional regulation, family functioning plays a buffering role in protecting the psychological well-being of pregnant women (7). It encompasses components such as emotional connections among family members, adaptability to changes, effective communication, and the capacity to manage life events (8). In Asian cultures heavily influenced by Confucian traditions, family functioning holds particular importance in supporting maternal mental health. Optimal family functioning not only ensures the effective fulfillment of members’ needs (9) but also provides emotional and practical support during stressful periods, alleviating depressive symptoms and promoting emotional stability in pregnant women (10).

Stressful life events represent a major risk factor for prenatal depression. They can trigger psychological distress, including anxiety and depression, in pregnant women and are associated with an increased likelihood of adverse pregnancy outcomes (11). These events typically require individuals to make adaptive adjustments and often pose potential risks or threats, acting as critical stressors that precipitate depressive symptoms (12). Stressful events may arise from various aspects of life, with family-related stressors being particularly salient during pregnancy. Recognizing the central role of family in maternal mental health, this study examines family-related stressful life events as key predictors of depressive symptoms during pregnancy.

Despite existing research focusing primarily on maternal characteristics and pregnancy intention as factors influencing prenatal depression, few studies have explored the specific impact of family functioning during the second trimester. While some research has investigated the individual and pairwise relationships among stressful life events, family functioning, and depression, there is limited exploration of the mechanisms linking these three factors. Compared to developed Western societies, the traditional family-oriented values, structures, and lifestyles in China often result in prolonged interactions between pregnant women and their family members (13). The family function of pregnant women will affect their ability to regulate their own emotions (7). According to the buffering effect model of social support (14), when a pregnant woman experiences a stressful life event, if she feels that the support from family members is strong enough, her negative cognition of the stressful life event will be alleviated, reducing or eliminating the stress response effect (10). This underscores the need to investigate the mediating role of family functioning in second trimester depressive symptoms.

To address this gap, the present study aims to comprehensively assess depressive symptoms, family functioning, and stressful life events among women in their second trimester of pregnancy. It seeks to unravel the complex interrelationships among these variables, with a specific focus on the mediating role of family functioning in the linkage between stressful life events and second trimester depressive symptoms. The findings are expected to inform the development of targeted mental health interventions for pregnant women, thereby reducing prenatal depressive symptoms, enhancing maternal and infant health, and promoting family harmony and societal well-being.

## 2 Materials and methods

### 2.1 Study population

This study is part of the large-scale, prospective Wuhan Healthy Baby Birth Cohort, conducted by the Wuhan Women’s and Children’s Health Care Center (WWCHCC), China. A total of 3386 pregnant women who underwent second trimester prenatal checkups at the center between April 2017 and February 2020 were recruited as the study population.

Participants were included based on the following criteria: 1. Voluntary participation and consent to follow-up; 2. Good general health, with no sever mental disorders; 3. Attendance at prenatal check-ups and delivery at the WWCHCC; 4. Ability to independently complete the questionnaires and communicate without barriers; 5. No history of psychiatric medication use or alcohol abuse.

After enrollment, trained staff conducted regular follow-ups, including physical examinations and questionnaire surveys, during early pregnancy, the second and third trimesters, delivery, and the postpartum period. Of the 3386 recruited participants, 3279 pregnant women met the eligibility criteria. Finally, 3279 women with complete data at a gestational age of 13–28 weeks were included in the analysis, resulting in an effective response rate of 96.8%.

All participants voluntarily provided written informed consent. The study was approved by the Ethics Committees of the WWCHCC (#2010009, 9 October 2012) and Huazhong University of Science and Technology ([2012]#14, 8 October 2012).

### 2.2 10-item Center for Epidemiologic Studies Depression Scale

Depressive symptoms in pregnant women were assessed using the 10-item Center for Epidemiologic Studies Depression Scale (CESD-10). This scale, developed by Andresen et al., is a simplified and revised version of the original CESD scale. The Chinese version of CESD-10 has been proven to have high reliability and validity (15). It has been widely used among Chinese adults and has satisfactory detection performance (16, 17). The scale comprises of 10 items, each rated on 4-point Likert scale with values ranging from 0 to 3. Among these, items 5 and 8 are scored in reverse. A cumulative score of ≥10 indicates the presence of depressive symptoms (18). In the current study, the Cronbach’s α coefficient for the scale was 0.654, demonstrating acceptable internal consistency.

### 2.3 Family APGAR Index

The subjective evaluation of overall family functioning was assessed using the Family APGAR Index, developed by Smilkstein in the United States (19). It is suitable for people of any age above teenagers and is widely used (20). This scale was introduced to China in 1995 by Lyu Fan and has demonstrated test-retest reliability ranging from 0.80 to 0.83. And the Chinese version has a good validation (21). It evaluates five dimensions: adaptability, partnership, growth, affection, and resolve. Each dimension is scored on a 3-point scale (2 = almost always, 1 = sometimes, 0 = almost never), with a total score ranging from 0 to 10. Higher scores reflect better family functioning. In this study, the Cronbach’s α coefficient for the questionnaire was 0.882, indicating good internal consistency.

### 2.4 18-item Stressful Life Events Scale

Stressful life events were evaluated using the standardized 18-item Stressful Life Events Scale, developed for the Hong Kong Family Cohort Study to investigate health, well-being, and family harmony. This scale can be used for pregnant women (22). This scale consists of five dimensions and a total of 18 items. Each item is scored on a scale of 0 to 10 based on the degree of impact. Higher scores indicate greater influence of stressful life events (23). In this study, the Cronbach’s α coefficient for the scale was 0.870, demonstrating good internal consistency.

### 2.5 Variables

The following covariates were selected as potential confounding factors in this study. Demographic characteristics were collected using a self-designed cohort questionnaire, including maternal age (<25, 25–29, 30–34, and ≥35 years), maternal educational level (high school or below, college or above), paternal educational level (high school or be-low, college or above), parity (primipara or multipara), and annual household income (<30,000, 30,000–49,999, 50,000–99,999, 100,000–199,999, ≥200,000 CNY).

In addition, certain lifestyle behaviors were included as covariates for analysis, such as second trimester sleep quality (very poor, poor, average, good, very good) and whether the pregnant woman continued working during pregnancy (yes or no).

### 2.6 Statistical analysis

Data analysis was conducted using SPSS version 26.0 and AMOS version 26.0 software. Descriptive statistics were conducted for demographic characteristics, with categorical data presented as frequencies and percentages, and continuous data expressed as means and standard deviations. Pearson and Spearman correlation analyses were used to examine the relationships among stressful life events, family functioning, and second trimester depression.

Multiple stepwise regression analysis was performed to explore factors influencing second trimester depression. The depression score was treated as the dependent variable, while variables from the demographic data, total family functioning score, and total stressful life events score were considered independent variables. Confounding variables were controlled during the analysis.

A structural equation model (SEM) was constructed using AMOS 26.0 to investigate whether the effect of stressful life events (independent variable) on second trimester depressive symptoms (dependent variable) was mediated by family functioning (mediator). Path analysis of latent variables was used to explore this potential mediating effect. The significance level was established at α = 0.01.

Finally, Bootstrap method was used to re-test the mediation effect. By resampling the original dataset (N = 3,279) 5,000 times, we estimated the indirect and direct effects of stressful life events on second trimester depression. The 95% confidence intervals that did not include zero were used to determine statistical significance.

## 3 Results

### 3.1 Demographic data

This study included 3279 pregnant women who completed the second trimester questionnaire from a birth cohort in Wuhan between April 2017 and February 2020. The majority of participants (82.9%) were between ages of 25 and 34. Most pregnant women (80.6%) had an educational level of college or above, and their husbands’ educational level was college or above for 80.2% of cases. Nearly half (43.6%) of the participants indicated an annual household income ranging from 100,000 to 200,000 CNY.

Additionally, the majority (69.7%) of the participants were primiparous, and most (62%) continued to work during pregnancy. Regarding sleep quality in the past month during the second trimester, 22.3% of participants reported poor sleep quality, 44.9% rated their sleep as average, and a total of 31.8% rated their sleep as good or very good. Meanwhile, 0.9% reported very poor sleep quality. Detailed data are presented in **Table 1**.

**Table 1:** Univariate analysis of demographic characteristics of pregnant women on second trimester depression and family functioning (N = 3279).

Variables	N (%)	Depressive symptoms		Family function		
		Absence (%)	Presence (%)	Sever dysfunction (%)	Moderate dysfunction (%)	Good (%)
Age (years)						
<25	158 (4.8)	115 (72.8)	43 (27.2)	14 (8.9)	37 (23.4)	107 (67.7)
25∼29	1549 (47.2)	1227 (79.2)	322 (20.8)	89 (5.7)	370 (23.9)	1090 (70.4)
30∼34	1169 (35.7)	916 (78.4)	253 (21.6)	81 (6.9)	284 (24.3)	804 (68.8)
≥35	403 (12.3)	309 (76.7)	94 (23.3)	45 (11.2)	108 (26.8)	250 (62.0)
*χ^2^*		4.214		18.740^**^		
*P*		0.239		**0.005**		
Maternal educational level						
high school or below	636 (19.4)	488 (77.6)	148 (22.4)	60 (10.2)	221 (33.5)	355 (56.3)
college or above	2643 (80.6)	2079 (78.7)	564 (21.3)	169 (6.4)	578 (21.9)	1896 (71.7)
*χ^2^*		4.022		69.858		
*P*		0.134		**<0.001**		
Paternal educational level						
high school or below	651 (19.8)	495 (73.2)	156 (26.8)	66 (12.3)	196 (33.8)	389 (53.9)
college or above	2628 (80.2)	2072 (78.8)	556 (21.2)	163 (6.2)	603 (22.9)	1862 (70.9)
*χ^2^*		6.443^*^		46.369		
*P*		**0.040**		**<0.001**		
Annual household income (CNY)						
<30,000	97 (3.0)	61 (62.9)	36 (37.1)	11 (11.3)	37 (38.1)	49 (50.5)
30,000–49,999	193 (5.9)	137 (71.0)	56 (29.0)	15 (7.8)	62 (32.1)	116 (60.1)
50,000–99,999	914 (27.9)	740 (81.0)	174 (19.0)	61 (6.7)	242 (26.5)	611 (66.8)
100,000–199,999	1431 (43.6)	1138 (79.5)	293 (20.5)	97 (6.8)	321 (22.4)	1013 (70.8)
≥200,000	644 (19.6)	491 (76.2)	153 (23.8)	45 (7.0)	137 (21.3)	462 (71.7)
*χ^2^*		26.312		30.722		
*P*		**<0.001**		**<0.001**		
Parity						
primipara	2285 (69.7)	1810 (79.2)	475 (20.8)	130 (5.7)	509 (22.3)	1646 (72.0)
multipara	994 (30.3)	757 (76.2)	237 (23.8)	99 (10.0)	290 (29.2)	605 (60.9)
*χ^2^*		3.804		44.208		
*P*		0.051		**<0.001**		
Second trimester sleep quality						
very good	106 (3.2)	94 (88.7)	12 (11.3)	8 (7.5)	25 (23.6)	73 (68.9)
good	939 (28.6)	790 (84.1)	149 (15.9)	54 (5.8)	206 (21.9)	679 (72.3)
average	1473 (44.9)	1138 (77.3)	335 (22.7)	112 (7.6)	375 (25.5)	986 (66.9)
poor	732 (22.3)	521 (71.2)	211 (28.8)	53 (7.2)	185 (25.3)	494 (67.5)
very poor	29 (0.9)	24 (82.8)	5 (17.2)	2 (6.9)	8 (27.6)	19 (65.5)
*χ^2^*		48.648		9.010		
*P*		**<0.001**		0.341		
Work while pregnant						
yes	2034 (62.0)	1615 (79.4)	419 (20.6)	95 (7.6)	333 (26.7)	817 (65.6)
no	1245 (38.0)	952 (76.5)	293 (23.5)	134 (6.6)	466 (22.9)	1434 (70.5)
*χ^2^*		3.912^*^		8.545^*^		
*P*		**0.048**		**0.014**		

* p<0.05, ** p<0.01.

Bold values indicate statistically significant results at p<0.05.

### 3.2 Prevalence of depression, family functioning, and stressful life events during the second trimester

Of all samples, 712 had depression, with a detection rate of 21.7%. Factors such as whether the pregnant woman continued working during pregnancy, the husband’s educational level, annual household income, and sleep quality during the second trimester in the past month were strongly associated with depressive symptoms (*P* < 0.05).

The total family functioning scores revealed that 229 participants (7.0%) experienced severe family dysfunction, 799 participants (24.4%) had moderate dysfunction, and 2,251 participants (68.6%) reported good family functioning. Maternal age, maternal educational level, paternal educational level, annual household income, parity, and whether the pregnant woman continued working during pregnancy all had significant impacts on family functioning (*P* < 0.05).

Among the 18 types of stressful life events, the three most commonly reported were: “Reduced social activities” (reported by 2305 participants, 70.3%), “Heavy workload” (reported by 1327 participants, 40.5%), and “A family member had serious health problems” (reported by 1004 participants, 30.6%). The least commonly reported event was “Being arrested or facing legal disputes,” which was reported by 114 participants (3.6%). Detailed results are presented in **Table 2**.

**Table 2:** Occurrence of stressful life events (N = 3279).

Dimensions	Stressful life events	Occurrence	N	Percentage (%)
Family and Household Dimension	Death of a family member	Yes	519	15.8
		No	2760	84.2
	Death of a close friend	Yes	191	5.8
		No	3088	94.2
	A family member had serious health problems	Yes	1004	30.6
		No	2275	69.4
	Relocation to a new residence	Yes	721	22.0
		No	2558	78.0
	Moving out by me or a family member	Yes	281	8.6
		No	2998	91.4
	Addition of a new family member	Yes	527	16.1
		No	2752	83.9
	Establishment of a new household	Yes	915	27.9
		No	2364	72.1
	Reduced family gatherings	Yes	1022	31.2
		No	2257	68.8
Personal and Social Dimension	Serious health issues of a close friend	Yes	314	9.6
		No	2965	90.4
	Being arrested or facing legal disputes	Yes	114	3.6
		No	3165	96.5
	Reduced social activities	Yes	2305	70.3
		No	974	29.7
	End of relationship with spouse/partner/boyfriend or girlfriend	Yes	153	4.7
		No	3126	95.3
	Beginning of a new romantic relationship	Yes	129	3.9
		No	3150	96.1
Health Dimension	Experiencing a serious health problem	Yes	491	15.0
		No	2788	85.0
	Experiencing physical violence or harm	Yes	123	3.8
		No	3156	96.2
Work Dimension	Heavy workload	Yes	1327	40.5
		No	1952	59.5
	Job loss or continued unemployment	Yes	710	21.7
		No	2569	78.3
Financial Status Dimension	Financial deterioration for me or my family	Yes	853	26.0
		No	2426	74.0

### 3.3 Correlation analysis of second trimester depression, family functioning, and stressful life events

Pearson and Spearman correlation analyses were used to examine the relationships among stressful life events, family functioning, and second trimester depression. Correlation analysis revealed a significant inverse relationship between family functioning and second trimester depression (*r* = -0.334, *P* < 0.001). Stressful life events were significantly positively correlated with second trimester depression (*r* = 0.165, *P* < 0.001). Additionally, family functioning and its subdimensions exhibited a significant negative correlation with stressful life events (*r* = -0.129, *P* < 0.01). Detailed results are presented in **Table 3**.

**Table 3:** Correlations between study variables.

Variable	Second trimester depression	Stressful life events	Family functioning
Second Trimester Depression	1		
Stressful Life Events	0.165**	1	
Family Functioning	-0.334**	-0.129*	1

* *P*<0.01. ** *P*<0.001.

The significant correlations between variables provide support for testing the mediating effect.

### 3.4 Mediating role of family functioning in the relationship between stressful life events and second trimester depression

First, multiple stepwise regression analysis identified the total family functioning score, total stressful life events score, poor sleep quality, average sleep quality, whether the pregnant woman continued working during pregnancy, and total household income as significant factors influencing second trimester depression.

Next, a mediation analysis was performed to examine the relationship between stressful life events and second trimester depression, with major demographic covariates controlled for. The results, as shown in **Table 4**, indicated that the direct predictive effect of stressful life events on second trimester depression was significant. After including family functioning as a mediating variable, this effect remained significant (c = 0.131, *P* < 0.001).

**Table 4:** Hypothesis testing of path analysis.

Path relationship	Standardized path coefficient	SE	*P*
Stressful Life Events → Second Trimester Depression	c=0.131	0.063	<0.001
Stressful Life Events → Family Functioning	a=-0.107	0.008	<0.001
Family Functioning → Second Trimester Depression	b=-0.343	0.160	<0.001

**Figure 1:**
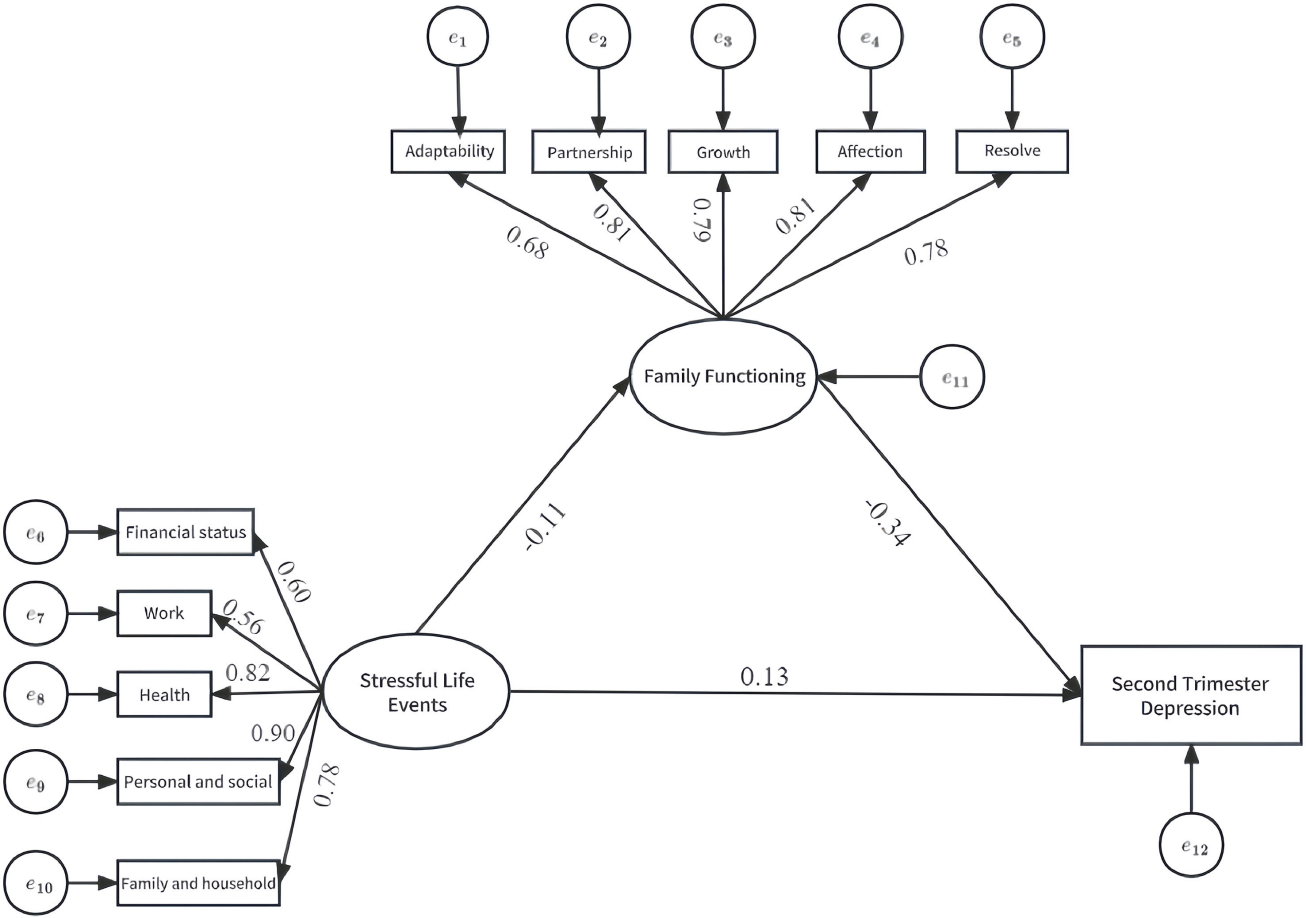
Standardized path coefficient.

In the indirect pathway, stressful life events found to be a significant negative predictor of family functioning (a = -0.107, *P* < 0.001), which in turn significantly predicted an increase in second trimester depression (b = -0.343, *P* < 0.001). According to the standardization coefficient, **Figure 1** can be plotted in AMOS.

The model fit indices were as follows: *χ²*/df = 10.802, CIF = 0.956, TLI = 0.947, RMSEA = 0.055. Due to the large sample size of this study, the χ²/df value, which is sensitive to sample size, is slightly higher. With the exception of this value, all other fit indices were within acceptable ranges, indicating that the proposed model was acceptable (24).

Additionally, the mediating effect was tested through the Bootstrap method. A total of 5,000 random samples were drawn from the original dataset (N = 3279), and the 95% confidence intervals were calculated. The results, as shown in **Table 5**, indicated that the Bootstrap 95% confidence interval for the indirect effect path did not include 0, confirming the presence of a significant mediating effect.

**Table 5:** Bootstrap analysis of the mediating effect of family function on the relationship between stressful life events and second trimester depression.

Path relationship	Effect	SE	95% CI
Stressful Life Events → Second Trimester Depression (indirect effect)	0.133	0.028	0.081~0.190
Stressful Life Events → Second Trimester Depression (direct effect)	0.476	0.077	0.328~0.628
Stressful Life Events → Second Trimester Depression (total effect)	0.608	0.083	0.451~0.774

Thus, family functioning partially mediated the association between stressful life events and second trimester depression, with the mediating effect accounting for 21.9% of the total effect.

## 4 Discussion

Our study found that 21.7% of pregnant women experienced second trimester depression, a prevalence rate consistent with prior research. In earlier cohort studies, the prevalence of second trimester depression in different regions of China ranged from approximately 19.7% to 23.7% (5, 25, 26). Although the tools used to assess depression, as well as the populations and regions studied, varied across these studies, the prevalence rates reported in large-sample cohort studies were relatively consistent. This suggests that antenatal depression is not specific to certain regions or populations but rather has risk factors that are common across regions. These findings underscore the importance of maternal and child healthcare professionals, as well as other relevant personnel, giving increased attention to this issue.

In our study, factors such as whether the pregnant woman continued working during pregnancy, her husband’s educational level, annual household income, and sleep quality in the past month all significantly influenced second trimester depression, which aligns with the results of other studies (27, 28). Pregnant women with better economic conditions are more likely to access high-quality healthcare services and are less likely to worry about material needs during pregnancy and child-rearing, which helps them maintain a positive mood. Additionally, some studies (29) have suggested that engaging in moderate hobbies, such as dancing, practicing yoga, or traveling, can reduce the incidence of depressive symptoms and alleviate depressive moods. These activities require financial support and leisure time. Pregnant women who continue working during pregnancy often have limited personal time and monotonous leisure activities, which can adversely affect their mental health.

Furthermore, in our study, maternal age, educational level, husband’s educational level, annual household income, parity, and whether the pregnant woman continued working during pregnancy significantly affected family functioning, consistent with findings from previous studies (10, 13). These findings suggest the importance of providing educational support and appropriate knowledge dissemination to pregnant women with lower cognitive levels or less favorable family conditions. Such efforts could help pregnant women and their families improve their emotional regulation skills and overall well-being.

Our study also confirmed the significant correlations among stressful life events, family functioning, and second trimester depression. The strong correlation between stressful life events and second trimester depression indicates that issues arising from various aspects of stressful life events elevate the risk of depression in pregnant women. Previous studies (30) have shown that due to changes in physical appearance and hormonal levels during pregnancy, pregnant women tend to be more emotionally vulnerable and sensitive, making them more susceptible to stress from life events, which in turn leads to depressive and anxious reactions. Additionally, excessive cortisol levels produced during stress adaptation are another risk factor that increases the likelihood of depression (31).

Family functioning also exerts a negative impact on antenatal depression. Family dysfunction serves as a risk factor for antenatal depression, meaning that the poorer the family functioning, the higher the risk of depression. Previous studies (32) have similarly demonstrated the association between family functioning and the prevalence of depressive symptoms. Good family functioning helps pregnant women maintain a positive mood and serves as a protective factor against antenatal depression. Pregnant women should be encouraged to communicate more with their family members, who, in turn, should provide greater care and understanding. Creating a positive family environment during pregnancy can promote better family function. Giving pregnant women more care and understanding can help them maintain a healthy mentality, reduce the occurrence of prenatal depression, and ultimately promote the health of mothers and babies.

Furthermore, previous studies have rarely explored a model involving stressful life events, family functioning, and depression symptoms among pregnant women in the second trimester. Our study used family functioning as a mediating variable to examine its role in the relationship between stressful life events and second trimester depression symptoms. The results confirmed the validity of the mediation model, indicating that beyond the direct effect of stressful life events on second trimester depression, stressful life events can also indirectly affect depression through family functioning, accounting for 21.9% of the total effect.

Through testing, it was found that stressful life events have a significant negative predictive effect on family function. After pregnant women experience a certain number of negative life events during and before pregnancy, their stress increases, especially in the three dimensions of family and household, personal and social and financial status. When facing stress, they feel a lack of support from their families and a lack of security, which causes emotional accumulation. After adding the mediating variable family function, the positive predictive effect of stressful life events on depression in the second trimester was still significant. In other words, family functions will further act on the mental health of pregnant women. Better family functioning increases satisfaction with family care, thereby reducing the likelihood of depression during the second trimester. Studies have also suggested that high life satisfaction under stressful circumstances can protect pregnant women’s mental and physical health, reducing the risk of depression. Since family care is a vital source of life satisfaction for pregnant women, improving family functioning undoubtedly serves as a safeguard for their mental and physical well-being. Therefore, from the perspective of family function, we should pay attention to the importance of family to pregnant women, including family partnership, adaptability, affection, growth, etc., and give pregnant women more care and understanding.

In summary, antenatal depression is relatively common among women in their second trimester. Prevention and intervention strategies should focus on reducing exposure to stressful life events and improving family functioning. Psychological health education during pregnancy should emphasize the importance of family care. Pregnant women and their families should be informed about relevant medical and healthcare knowledge. Additionally, spouses and other family members should be encouraged to provide more support and companionship during pregnancy, helping expectant mothers feel cared for and valued. Special attention should be given to women with impaired family functioning, offering targeted psychological interventions when needed. Furthermore, family-based prenatal education should be provided to enhance communication among family members and foster a supportive home environment for the pregnant woman. Enhancing family functioning both directly and indirectly can improve pregnant women’s ability to regulate negative emotions, thereby reducing the occurrence of depression in the second trimester and ensuring maternal and fetal health.

This study is based on a large birth cohort and uses standardized measurement tools. The findings are prospective and relatively robust. It enhances the mechanistic understanding of prenatal depression and provides a theoretical basis for developing more comprehensive interventions. However, our study has several limitations. For instance, all questionnaires were self-reported by pregnant women, relying on subjective retrospective assessments that may be subject to recall bias and could potentially affect the results. Moreover, family functioning does not fully mediate the relationship between stressful life events and depression during the second trimester. Other factors, such as social support (33), may also play a role in this association. Future research should further explore these factors to achieve a more comprehensive understanding. From a public health perspective, this research could be extended to other regions. Multi-center or longitudinal studies would help clarify the causal relationships among stressful life events, family functioning, and prenatal depression. Such efforts would enhance the clinical relevance of the findings.

## Data Availability

The raw data supporting the conclusions of this article will be made available by the authors, without undue reservation.
